# Characterization of monoclonal antibodies against porcine epidemic diarrhea virus S1/S2 junction protein

**DOI:** 10.1186/s13568-023-01573-4

**Published:** 2023-07-12

**Authors:** Nan Huang, Qiaoli Lang, Liping Li, Liangpeng Ge, Xi Yang

**Affiliations:** 1 Chongqing Academy of Animal Sciences, Chongqing, 402460 China; 2 National Center of Technology Innovation for Pigs, Chongqing, 402460 China; 3 Key Laboratory of Pig Industry Sciences, Ministry of Agriculture, Chongqing, 402460 China

**Keywords:** Porcine epidemic diarrhea virus, Monoclonal antibodies, Antibody subclasses, Isotype identification method

## Abstract

Pig producers have faced considerable economic losses due to porcine epidemic diarrhea virus (PEDV) infection, emphasizing the need for PEDV antibody development. The S1/S2 junction (S1S2J) cleavage site of the S protein of PEDV is one of the major determinants of coronavirus infection success. In this study, we specifically selected the S1S2J protein of PEDV-AJ1102 (a representative strain of the G2 type) as a target protein to immunize mice and generated monoclonal antibodies (mAbs) using hybridoma technology. Three mAbs with high-binding activities to the S1S2J protein and were obtained and further analyzed. To reveal the characterization of these mAbs, variable region genes of antibodies were studied by using DNA sequencing, thereby revealing differences in their CDR3 amino acid sequences. We then developed a new method to identify the isotypes of these three mAbs. Results showed that these three antibodies were of the IgM type. As for the functions of these three mAbs, indirect immunofluorescence assay confirmed their good binding ability to Vero E6 cells infected with the PEDV-SP-C strain (G1 type). Epitope analysis showed linear epitopes for all three mAbs. These antibodies were also used to detect infected cells via flow cytometry analysis. In summary, we prepared and examined three mAbs against PEDV-S1S2J. These mAbs can be employed as detection antibodies for diagnostic reagents and further developed for other applications. We also designed a novel technique for easy and cost-saving identification of isotypes of mouse mAbs. Our results lay a good foundation for the development of research on PEDV.

## Key points

- The S1S2J protein was used for the first time as a target antigen to develop PEDV mAbs
- Three PEDV mAbs that bound to PEDV S1/S2 junction region were generated. The functions of these three PEDV mAbs were evaluated by EC50, FCAs, and IFA
- This work developed a new method for easy and cost-saving identification of isotypes of mouse mAbs

## Introduction

Porcine epidemic diarrhea, which is caused by the porcine epidemic diarrhea virus (PEDV), affects swine of all ages (Jung et al. 2020; Li et al. 2020). This disease is characterized by vomiting, acute diarrhea, and dehydration and has high mortality in neonatal piglets (Debouck and Pensaert 1980). PEDV was first reported in England (Wood 1977) about 30 years ago. Unfortunately, an increasing number of mutations in PEDV have been identified (Lin et al. 2016). Pig producers have suffered serious economic losses as a result of PEDV.

As a member of the *Coronaviradae* family, PEDV is an enveloped single-strand RNA virus with a spike (S) protein on its surface (Hou et al. 2007) which can induce virus-neutralizing antibodies (Chang et al. 2002; Kirchdoerfer et al. 2021; Li et al. 2017; Song et al. 2016; Supekar et al. 2004). PEDV continues to evolve rapidly, with many S gene variants emerging around the world. PEDV strains can be divided into genogroup 1 (G1) and G2 according to their S protein variations (Guo et al. 2019; Wang et al. 2019). The G1 type includes classical strains (e. g. prototype strain CV777), and the G2 type contains varied insertion and deletion mutations in the S gene that are prevalent in different regions globally. These mutations may prevent many currently available commercial methods based on anti-S protein antibodies from successfully providing effective diagnostic functions or protection against PEDV variations. Therefore, the development of antibodies that can be used for comprehensive research on PEDV infection is crucial.

The coronavirus S protein undergoes protease cleavage in two domains, S1 and S2 (de Haan et al. 2008; Sun et al. 2007). The protease-mediated entry in the S1/S2 junction (S1S2J) cleavage site is one of the major determinants of coronavirus infection success (Matsuyama et al. 2005). Moreover, S1S2J is regarded as a relatively conserved region in the S protein.

In this study, we utilized the recombinant PEDV S1S2J protein (residues 630–800 of the S protein of PEDV strain AJ1102 [G2 type], a PEDV vaccine strain used widely in China) expressed by Expi293 suspension cells to immunize BALB/c mice and screened antibodies by using the PEDV-SC-P strain (G1 type). We prepared and evaluated three mAbs against PEDV-S1S2J. These mAbs can be used as detection antibodies for diagnostic reagents and further developed for other applications. We also developed a new technique for easy and cost-saving identification of isotypes of mouse mAbs. Our results lay a good foundation for the development of research on PEDV.

## Materials and methods

### Cell, virus, and mouse

The Vero E6 cell (Procell Life Science &Technology Co., Ltd, China) was cultured in Dulbecco’s Modified Eagle Medium (DMEM; Procell Life Science &Technology Co., Ltd, China) supplemented with 10% fetal bovine serum (FBS; Biological Industries, Israel) and 1% penicillin–streptomycin solution (Procell Life Science &Technology Co., Ltd, China). Three mice were purchased from Beijing Vital River Laboratory Animal Technology Co., Ltd. All animal experiments were approved by the Committee on the Ethics of Animal Experiments of Chongqing Academy of Animal Sciences. The PEDV-SP-C strain was propagated and titrated in Vero E6 cells (Zhao et al. 2015).

### Generation of full-length and truncated PEDV-S1S2J proteins

The S1S2J gene fragment (630-800aa) of spike protein gene sequences from the PEDV AJ1102 strain (GenBank accession no. AFQ37598.1) was optimized, artificially synthesized, and cloned into the pcDNA3.4 vector (Invitrogen, USA) to generate the S1S2J protein with a C-terminus mouse IgG2a Fc tag and a N-terminus 10 × His tag. After transient expression in Expi293 suspension cells (Thermo Fisher Scientific, USA), the S1S2J fusion protein was enriched using a HisTrap HP column (GE Healthcare, USA) according to the manufacturer’s guidelines.

To determine the epitopes, the gene fragment of PEDV S1S2J was truncated into three segments with 45 bp overlap between neighboring parts and cloned into the pcDNA3.4 vector. The plasmids expressing truncated proteins fused to the Fc part of mouse IgG2a at the C-terminus were transiently expressed in Expi293 suspension cells and enriched using a HiTrap Protein A HP affinity purification column. Protein concentration was ascertained using the A280 absorption value of Nanodrop2000 (Thermo Fisher Scientific, USA).

### SDS-PAGE and western blotting analysis

The protein samples were mixed well with sulfate–polyacrylamide gel electrophoresis (SDS-PAGE) sample buffer and subjected to 12% SDS-PAGE electrophoresis. For SDS-PAGE staining, the protein gels were visualized by using Coomassie brilliant Blue staining (Beyotime, China). For Western blotting analysis, the proteins were transferred electrophoretically to a 0.45 μm PVDF Transfer Membrane (Thermo Fisher Scientific, USA). After blocking in 5% skim milk for 2 h, the membranes were incubated with Alexa Fluor 680 donkey anti-mouse IgG (H + L) Highly Cross-Adsorbed Secondary Antibody (Invitrogen, USA) (1:5000) for 2 h at room temperature. After being washed 3 times with PBST, the membranes were imaged using the Odyssey^®^ CLX platform.

### Preparation of PEDV-S1S2J antibodies

The method employed in this work is from our previously published paper (Yang et al. 2022). Briefly, three female BALB/c mice (6 weeks old) were immunized subcutaneously with 100 μg purified PEDV S1S2J protein in Freund’s complete adjuvant (Sigma-Aldrich, USA). Three booster immunizations were performed at 2 week intervals with the same dose of S1S2J protein in Freund's incomplete adjuvant (Sigma-Aldrich, USA). The antibody titer of immunized mice serum was detected by enzyme-linked immunosorbent assay (ELISA) analysis. At 2 weeks after the final booster immunization, a final intraperitoneal immunization of the S1S2J protein was administered without an adjuvant. After 3 days, the spleen cells were isolated and fused to SP2/0 myeloma cells at a ratio of 5:1 using a bipolar pulsed electric field electrofusion method (Ke et al. 2019). Hybridoma cells were screened and monoclonal by using the ClonaCell^™^-HY Hybridoma Cloning Kit (STEMCELL Technologies Inc). Positive clones were selected by ELISA coating with the S1S2J protein.

### ELISA

An indirect ELISA was performed to ascertain the antibody titer of the immunized mice serum or positive hybridoma cells. Briefly, the purified full-length S1S2J protein (2 µg/mL) was used to coat 96-well plates overnight at 4℃. After being washed 3 times with PBST, the plates were blocked with 2% BSA for 2 h at 37 °C. Then, diluted serum or hybridoma supernatant was added to plates and incubated for 1 h at 37 °C. After three washes, a 1:5000 dilution of anti-Mouse IgG (Fab specific)-peroxidase antibody produced in goat (Sigma-Aldrich, USA) was added to plates and incubated for 1 h at 37 °C. After five washing steps, the reacting results were visualized by using TMB (3,3′,5,5′-tetramethylbenzidine, Solabio), and stopped via the addition of 2 mol/L H_2_SO_4_. Absorbance at 450 nm was determined using a microplate reader (Epoch, BioTek Instruments, Inc.).

### DNA sequencing of antibodies

The total RNA of hybridoma cells was isolated and the cDNA was synthesized with the PrimeScript^™^ One Step RT-PCR Kit (Takara, Japan). The target gene was amplified by PCR and analyzed by Sanger sequencing. DNA sequencing of the light chain of antibodies was determined using a previously described method (Yan et al. 2017). To detect the isotypes of the mouse immunoglobulin heavy chain, we designed several pairs of primers for the amplification. As shown in Table 1, the forward primers of the mouse immunoglobulin heavy chain were located in a signal peptide of a variable region, and the reverse primers were located in the CH2 regions of IgA, IgE, IgG, IgM, or CH3 region of IgD. The subtypes of antibodies were detected by PCR with forward primers mixture and a reverse primer. After sequencing the PCR products, subclasses analysis of the antibodies was performed using the BLAST program.

**Table 1:** Primers

Primer names	Sequences (5’-3’)
mVH-F-1	ATGGRATGSAGCTGKGTMATSCTCTT
mVH-F-2	ATGRACTTCGGGYTGAGCTKGGTTTT
mVH-F-3	ATGGCTGTCTTGGGGCTGCTCTTCT
mVH-F-4	CAGTCTGGACCTGAGCTGAAGAAGCCT
mVH-F-5	CAGTCTGGACCTGAGCTGGTGAAGCCT
mIGHA_CH2-R	CAGCTTTCTTCTGCACTGCATCCTT
mIGHE_CH2-R	GGTGAAGGTGCTTTCAGACATCCATT
mIGHG_CH2-R	ACCYTGCATTTGAACTCCTTGCC
mIGHM_CH2-R	CAGGTTCAGCCAGTCGATTTCAGAGA
mIGHD_CH3-R	CTGGTAGTCTCAGGACACTCCAGGT

### Indirect immunofluorescence assay (IFA)

After infection with PEDV strains, the obvious cytopathic effect (CPE) of Vero E6 cells was observed at 48 h. Then, the cells were fixed with 4% paraformaldehyde at room temperature for 30 min, followed by blocking with 5% BSA at 37 °C for 1 h. Each hybridoma supernatant was added and incubated overnight at 4 °C. After three washes, the cells were stained with goat anti-mouse IgG H&L (FITC) (1:1000 dilution) (Abcam, UK), followed by incubation for 1 h at 37 °C. Images of the stained cells were then visualized using an inverted fluorescence microscope (DMi8, Leica Microsystems).

### Flow cytometry analysis

Monocellular suspensions of the PEDV-infected Vero E6 cells were obtained after infection with PEDV strains for 48 h and incubated with hybridoma supernatant on ice for 1 h. After washing 3 times with 2% FBS-PBS (2% FBS in PBS), the cells were stained with goat anti-mouse IgG H&L (FITC) (1:1000 dilution) (Abcam, UK) for 30 min at 4 °C. The cells were washed 3 times, resuspended in 2% FBS-PBS, and detected by BD FACSVerse^™^ Flow Cytometer. The resulting data were analyzed with BD FACSuite^™^ v1.0.6 software.

### Statistical analysis

The GraphPad Prism 6.0 software was used for data analysis and processing.

## Results

### Expression and purification of recombinant PEDV-S1S2J protein

**Fig. 1:**
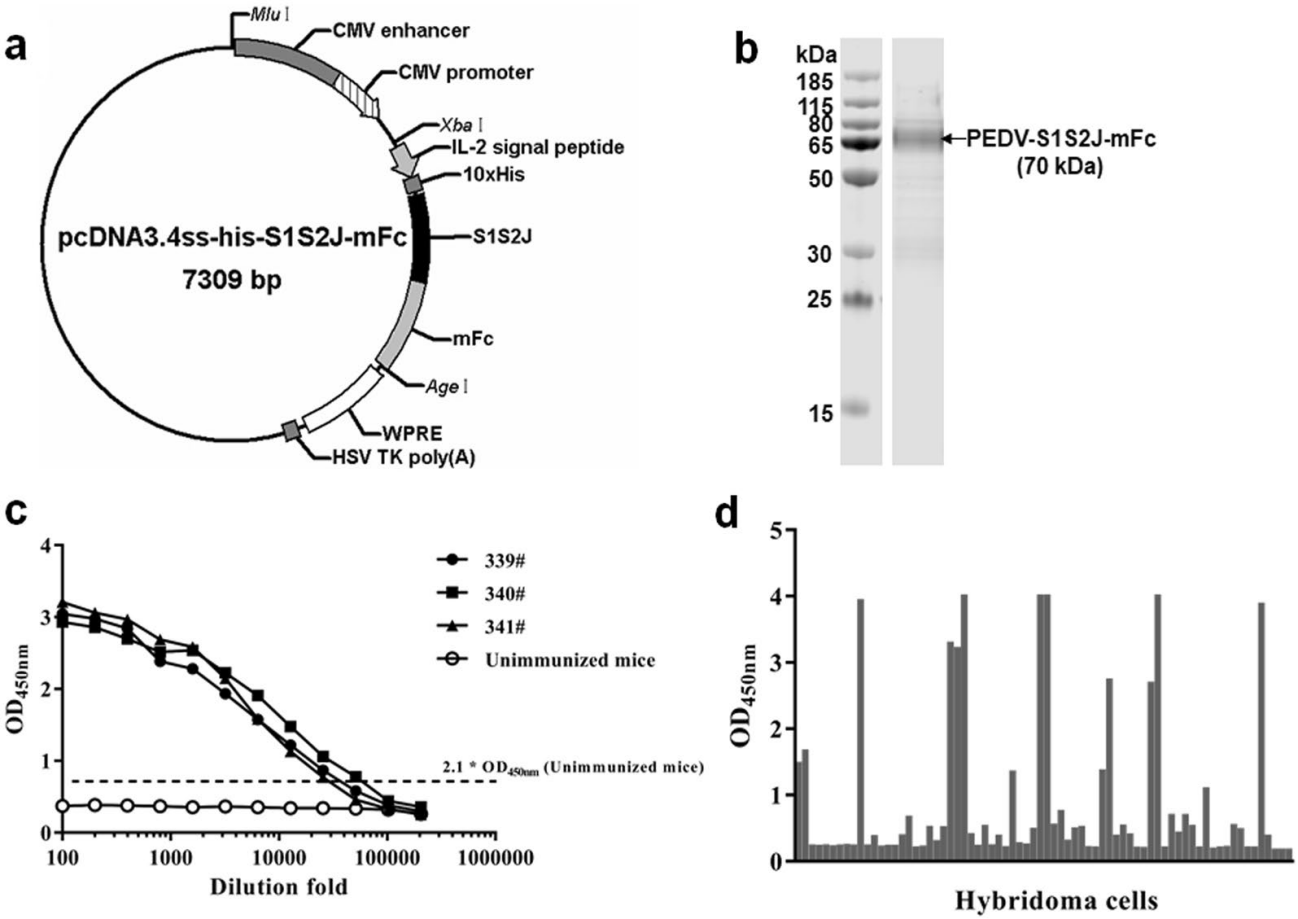
Preparation of PEDV-S1S2J antibodies. **a** The recombinant vector pcDNA3.4ss-his-S1S2J-mFc containing the cytomegalovirus (CMV) enhancer region (CMV enhancer), CMV promoter, human interleukin-2 (IL-2) signal peptide, 10 × His, S1S2J gene fragment (630–800aa) of spike protein gene sequences from PEDV AJ1102, mouse IgG2a Fc gene (mFc), woodchuck hepatitis post-transcriptional regulatory element (WPRE) and HSV TK poly (A) was prepared for expression of recombinant PEDV-S1S2J protein in Expi293 cells. **b** The recombinant PEDV-S1S2J protein was purified by using a HisTrap HP column and analyzed by SDS-PAGE. BALB/c mice were immunized with recombinant PEDV-S1S2J protein 4 times. **c** The serum titers of the immunized mice were detected by ELISA analysis. Splenocytes prepared from immunized mice were fused with SP2/0 cell. **d** Hybridoma cells were screened by ELISA for the presence of antibodies against the S1S2J protein

To express the PEDV-S1S2J protein, the S1S2J gene with the human IL-2 signal peptide, a mouse Fc tag (mFc), and a 10 × His tag was inserted into the eukaryotic expression vector pcDNA3.4, leading to generation of plasmid pcDNA3.4ss-his-S1S2J-mFc (Fig. 1a). The recombinant S1S2J protein was expressed in the Expi293 expression system and purified with a HisTrap HP column. As shown in Fig. 1b (SDS-PAGE analysis), we successfully obtained high-purity (> 90%) recombinant S1S2J protein with a 70 kDa molecular weight. The majority of the recombinant S1S2J protein was present in monomer form (70 kDa), and a small amount of protein (< 10%) was present at the positions of ~ 140 kDa and ~ 30 kDa, thereby implying that the recombinant S1S2J protein forms a dimer and an mFc fragment.

### Preparation and identification of PEDV-S1S2J antibodies

Three female BALB/c mice were immunized with recombinant PEDV-S1S2J protein. All serum titers of the three mice exceed 1:10,000 after 5 times of immunization (Fig. 1c). The hybridoma cells were obtained by the electrofusion method and screened for the presence of antibodies against the S1S2J protein through ELISA analysis. A total of 14 hybridoma clones were screened for their high binding activity (OD_450nm_ > 1.0) (Fig. 1d). To obtain stable hybridoma clones, we passed these 14 hybridoma clones for up to 20 generations and generated three hybridoma clones that stably secrete antibodies, denoted as 3-5G, 7-9D, and 8-3B.

**Fig. 2:**
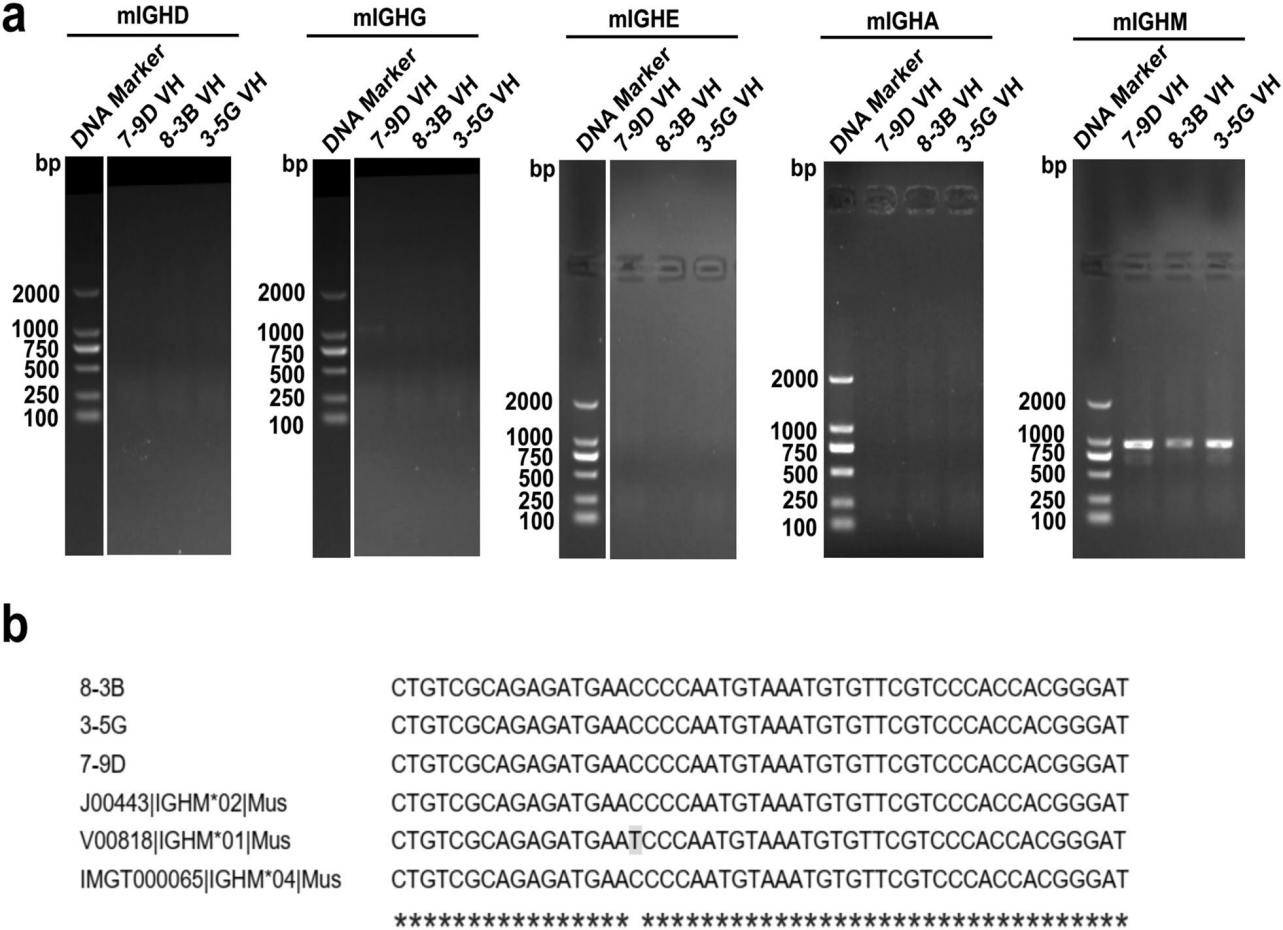
Identification of antibody subtype. Three hybridoma cell lines that stably secrete PEDV-S1S2J antibodies were generated in this study. **a** The subtypes of these antibodies were identified by PCR analysis. **b** Sequence alignment was performed on the PCR products

To identify the isotypes of these three antibodies, the total RNA of hybridoma cells was isolated, and cDNA was synthesized using T7 Oligo (dT) Prime. PCR results showed that all three clones were mouse antibodies of the IgM class (Fig. 2a). Furthermore, multiple sequence alignment after sequencing confirmed that the heavy chain gene of these three antibodies was the IGHM*02 allele or IGHM*04 allele of mouse IgM but not the IGHM*01 allele (Fig. 2b).

By using GenBank and the IMGT/ V-Quest database, we analyzed the hypervariable regions CDR1, CDR2, and CDR3 of these three antibodies (Table 2). The light chain CDR regions of the three antibodies significantly varied. Although 3-5G and 7-9D shared common sequence motifs in the CDR1 and CDR2 regions of the heavy chain, the CDR3 regions were significantly different. The antibody CDR3 is the most important region for antigen binding and its amino acid sequence has the most significant diversity among these CDRs. Therefore, 3-5G, 7-9D, and 8-3B were different monoclonal antibodies (mAbs).

**Table 2:** Antibody variable region analysis

	Locus	CDR1	CDR2	CDR3
3-5G_VH	IGHV1-26*01	GYSFTGYT	INPYNGGT	ARGGVLWLRRGNYFDY
3-5G-VK	IGKV4-91*01	SSISSNY	RTS	QQGSSIPLT
8-3B-VH	IGHV1-87*01	YTFSR	GQGLEWIG	SEDSAVYYCAFMDY
8-3B-VK	IGKV4-57*01	SSVSY	STS	QQRSSYPFT
7-9D-VH	IGHV1-26*01	GYSFTGYT	INPYNGGT	ARIHYYGYVGYFDY
7-9D-VK	IGKV8-21*01	QSLLNSRTRKNY	WAS	KQSYNLYT

### Epitope analysis

**Fig. 3:**
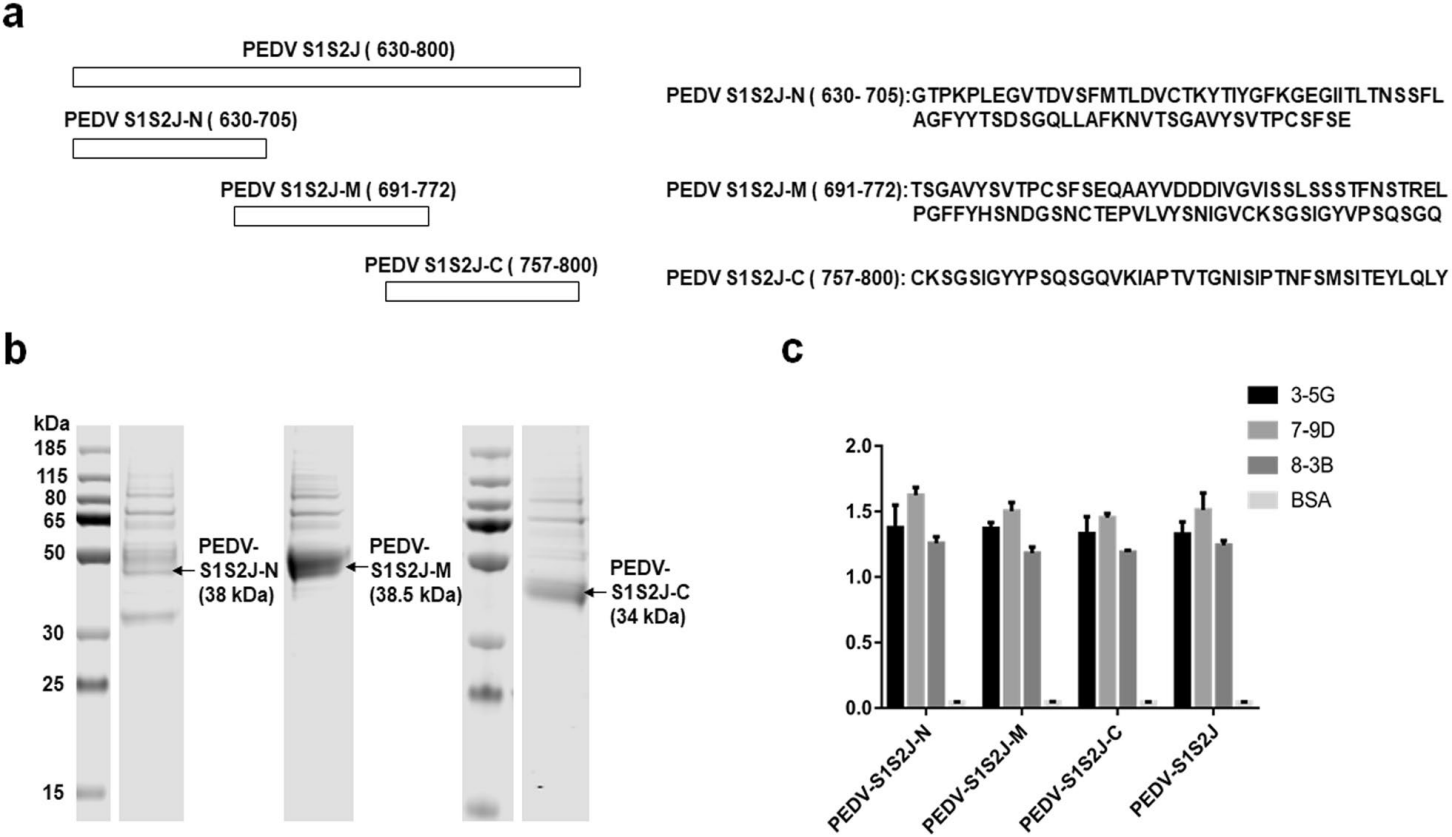
Epitope mapping of PEDV-S1S2J antibodies. **a** The full-length PEDV-S1S2J protein was truncated into three segments (designated as PEDV-S1S2J-N, PEDV-S1S2J-M and PEDV-S1S2J-C) with 15-amino acid overlap between neighboring parts. **b** These three truncated proteins were expressed in the Expi293 expression system, purified by a HiTrap Protein A HP affinity purification column and identified by Western blot using anti-mouse IgG antibody. **c** The purified truncated proteins were used as the coating antigen in ELISA analysis to detect the epitopes of 3-5G, 7-9D, and 8-3B mAbs

To study the epitopes of these three antibodies, the PEDV-S1S2J protein was truncated into three segments (designated as PEDV-S1S2J-N, PEDV-S1S2J-M, and PEDV-S1S2J-C). Each segment had an overlap of 15 amino acids (Fig. 3a). These three truncated PEDV-S1S2J proteins were expressed by the Expi293 Expression System and then purified by HiTrap Protein A HP column affinity chromatography (Fig. 3b). ELISA results revealed that 3-5G,7-9D and 8-3B showed high binding activity with the full-length and truncated PEDV-S1S2J proteins, but no binding activity was observed with the unrelated protein BSA (Fig. 3c).

### Functional analysis

**Fig. 4:**
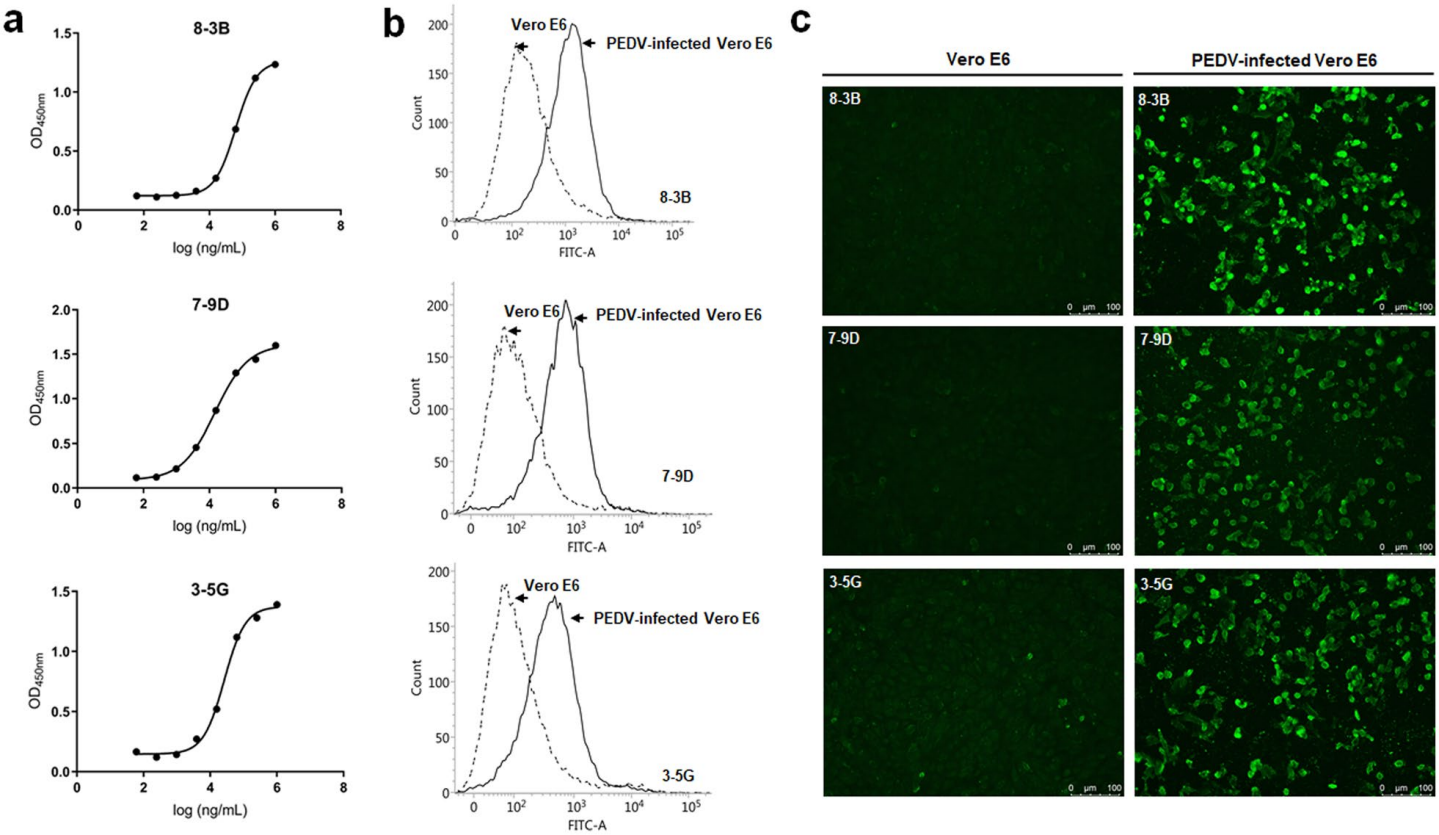
Function analysis of PEDV-S1S2J antibodies. **a** Determination of the antibody concentration for half-maximal binding (EC_50_) with the S1S2J protein using ELISA analysis. To ascertain the binding affinities of these three antibodies to infected cells, Vero E6 cells were infected with PEDV-SP-C strain (G1 type) for 48 h. PEDV-infected cells were detected by PEDV S1S2J antibodies; **b** flow cytometry analysis; and **c** immunofluorescence assay

The PEDV S1S2J protein expressed in this study belongs to a part of the S protein of the PEDV strain AJ1102 (G2 type), one of the PEDV vaccine strains used widely in China. We performed indirect ELISA to investigate the antibody concentration for half-maximal binding (EC_50_) with the PEDV S1S2J protein. As shown in Fig. 4, the 3-5G,7-9D and 8-3B mAbs showed high-binding activities with S1S2J protein in ELISA analysis, with EC_50_ values of 26.293, 14.337, 62.984 μg/mL respectively (Fig. 4a).

To analyze the binding activities of the 3-5G, 7-9D, and 8-3B mAbs to strains categorized under the PEDV G1 type, we used FCA and IFA to analyze their binding ability to cells infected with PEDV-SP-C strain (G1 type). FCA revealed that these antibodies bound significantly to Vero E6 cells infected with PEDV and could be used to distinguish between uninfected and PEDV-infected Vero E6 cells (Fig. 4b). In the IFA, all three antibodies could not bind to normal Vero E6 cells, but could bind to PEDV-infected Vero E6 cells (Fig. 4c). Among these antibodies, 8-3B showed the strongest GFP signals from immunofluorescence staining. All results implied that these three antibodies generated in our study were functional and useful tools for analyzing PEDV infection.

## Discussion

PEDV infection is a contagious intestinal disease in pigs with a high incidence rate in all ages and high mortality in suckling piglets. Moreover, PEDV has caused serious economic losses to the swine breeding industry (Turlewicz-Podbielska and Pomorska-Mol 2021). Similar to other coronaviruses, the S glycoprotein of PEDV is the major target for immune responses and mediates virus-cell attachment, receptor binding, virus-host membrane fusion, and entry. The S1/S2 junction site of S protein plays a key role in protease-mediated entry, is one of the determinants of success in coronavirus infection, and is a relatively conserved region in the S protein (Gobeil et al. 2021; Matsuyama et al. 2005). Therefore, the development of antibodies against S1S2J that can be used for comprehensive research on PEDV infection is vital.

To generate mouse antibodies that recognize both G1 and G2-types stains, we immunized mice with the S1SJ protein of PEDV-AJ1102 (a representative strain of G2 type) and selected antibodies by functional analysis with strain PEDV-SC-P (G1 type). The S1S2J protein was expressed by Expi293 suspension cells. For eukaryotes, the Expi293 expression system has many advantages, such as correct folding of higher-order structures, signal peptide cleavage, glycosylation, phosphorylation, oligomerization, and features that are critical for the S1S2J antigen. For the convenience of purification, we fused the codon-optimized sequence of the S1S2J gene to a C-terminus mouse IgG2a Fc tag and an N-terminus 10 X His tag in the pCDNA3.4 vector. As expected, we generated a high pure (> 90%) S1S2J protein (Fig. 1).

Three mAbs of PEDV were generated in this study. Note that variable region genes and antibody isotypes are vital for monoclonal antibodies. We also developed a novel technique for the easy and cost-saving identification of isotypes of mouse antibodies. Unlike commercially available mouse monoclonal antibody isotyping kits (such as the Rapid Isotyping Kit-Mouse, Thermo Scientific), our method only needs an additional 10 primers for antibody variable region genes sequencing.

Epitopes play important roles in inducing antibody production and cell-mediated immune response against pathogens. Therefore, identifying the antigenic epitopes of antibodies is essential for developing epitope-based vaccines and diagnostics. We also performed epitope analysis using three truncated PEDV-S1S2J proteins (Fig. 3). Antigenic epitopes always contain approximately 5–15 aa and include linear epitopes and spatial epitopes (Berger and Lapthorn 2016). However, most antigenic epitopes are spatial epitopes. Linear epitopes are continuous fragments of protein in the primary structure. In contrast, spatial epitopes are formed by neighboring amino acid residues on the surface of protein but are discontinuous in the primary protein structure (Burton 2002). As shown in Fig. 4c, 3-5G, 7-9D, and 8-3B displayed high protein binding activity of full-length and truncated PEDV-S1S2J proteins. These results indicated that the epitopes of 3-5G, 7-9D, and 8-3B are all linear.

The virus neutralization activities of 3-5G, 7-9D, and 8-3B were also detected in this work, and none of the antibodies prevented PEDV infection of Vero E6 cells (data not shown). However, non-neutralizing antibodies can also inhibit PEDV infection in vivo via indirect mechanisms dependent on the Fc receptor, such as antibody-dependent cellular cytotoxicity (ADCC), complement-dependent cytotoxicity (CDC) and antibody-dependent cellular phagocytosis (ADCP) (Abreu-Mota et al. 2018; Jegaskanda et al. 2013; Vogt et al. 2011). Therefore, 3-5G, 7-9D, and 8-3B can also be used as effective tools for research on PEDV infection.

In conclusion, we generated a high pure PEDV-S1S2J protein and developed three high-affinity monoclonal mouse IgM antibodies against this protein. These antibodies can be used as effective tools to facilitate the development of research on PEDV infection, immune responses, and diagnostic investigation. We also developed a new method for easy and cost-saving identification of isotypes of mouse mAbs. Our results lay a good foundation for the development of research on PEDV.

## Data Availability

The data reported in this paper have been deposited in the OMIX, China National Center for Bioinformation/Beijing Institute of Genomics, Chinese Academy of Sciences (https://ngdc.cncb.ac.cn/omix). The accession number for the nucleotide sequence of the optimized, artificially synthesized S1S2J gene fragment is OMIX002917. The accession number for variable region nucleotide sequences of PEDV S1S2J antibodies is OMIX002787.
